# 

**Published:** 1885-03-20

**Authors:** 


### TABLE OF CONTENTS.

ORIGINAL AND SELECTED ARTICLES:

Operative Procedure for Occluded Gall bladder, an Autopsic Lecture in the amphitheatre of the Southern Medical College, Atlanta, Ga., February 5th, 1885, by J. McF Gaston, M D., Professor of the Principles and Practice of Surgery, 81. Hydrochlorate of Cocaine as a Local Anæsthetic, by W. L Bullard, of Columbus, Ga., 88 New York Obstetrical Society, 89 Electricity in Diseases of the Nose, Throat and Ears, and in some diseases of the Thoracic and Abdominal Viscera which result from Chronic nasal and Pharyngo-nasal Catarrh, 94. Case of Abscess of the Lung Cured by Incision and Drainage, 97.

ABSTRACTS AND GLEANINGS:

Pneumonia Treated with Jaborandi, 100. Dugas' Bandage for the Breast, 102. Two New Methods for the Recognition of Albumen and Sugar in the Urine, 103. A Few Points of Diagnosis; The Present Status of the Germ Theory, 104 Pilocarpine in Erysipelas, 105. Rapid Recovery after Wound of Stomach and Protrusion of Viscera; Health of Children in Los Angeles; Kola, 106 Osmic Acid in Sciatica; Soluble Elastic Capsules of Quinine Sulphate, 107 Mangifera Indica; Thallin, the New Antipyretic, 108 Gangrene from a Hypodermic Injection of Quinine; Eserine, 109. Action of Salicylate of Soda Upon the Uterus; Spermatorrhœa—Monobromide of Camphor; Nitrate of Silver for Fissure of the Anus, 110.

SCIENTIFIC ITEMS:

System for Steering Balloons and Maintaining a Desired Elevation in the Atmosphere; Iridium, 111. What Fossils Teach; The Electric Light as a Scarf Pin; Meteoric Dust, 112.

PRACTICAL NOTES AND FORMULÆ.

A Local Application for Elongated Uvula; The Treatment of Dysentery with Iodide of Phenol; Some Good Formulæ, 113. Treatment of Neuralgia; For Wakefulness; Lactate of Quinine; An Ointment for Eczema of the Eyelids; For Bleeding Hemorrhoids, 114.

EDITORIALS AND MISCELLANEOUS.

Notice to Subscribers; Editorial Notices, 115, 116. Southern Medical College Commencement, 116. Books and Pamphlets Received; Receipted, 119. Special Notices, 120.

